# How earthquakes organize stress

**DOI:** 10.1073/pnas.2530754123

**Published:** 2026-02-06

**Authors:** Emily E. Brodsky, Gaspard Farge

**Affiliations:** ^a^Department of Earth and Planetary Sciences, University of California, Santa Cruz, CA 95060; ^b^Laboratoire de Géologie, Département de géosciences, École normale supérieure, Université Paris Sciences & Lettres, CNRS, Paris 75005, France

**Keywords:** earthquakes, friction, stress

## Abstract

Stress is not uniform in the Earth. Therefore, we must use natural experiments to measure the distribution of stresses and related quantities, rather than single values. For instance, dynamic triggering shows that faults are uniformly distributed over their loading cycles in Southern California. The probability that a fault ruptures across a barrier measures the in situ energy distribution. Fault roughness reflects the distribution of strength. These natural experiments produce observable distributions that are surprisingly consistent and suggest some degree of self-organization in the Earth’s crust. Once established, the functional form of the distributions can be used to track changes in response to earthquakes as well as to distinguish fundamentally different fault systems. Transient fault locking before stress release in laboratory experiments can be interpreted as a consequence of self-organization of fault stress. The robust self-organization of multiple variables in earthquake systems suggests that the most consequential mechanical outcome of earthquakes may be the redistribution of stress and the strain energy associated with it. The low friction on a fault during seismic slip as inferred by temperature measurements of the Tohoku earthquake is consistent with dissipation playing a secondary role to this redistribution process. Through stress redistribution and interaction, subduction zone faults tend to synchronize, perhaps due to their geometric simplicity, while the continental system of Southern California cannot synchronize, perhaps due to the complexity of the fault network. Earthquakes organize stress in the crust and produce a suite of well-defined, consistent distributions.

## Significance

Earthquakes organize the stress in the crust by redistributing it through slip events. As a result, fault systems evolve to preferred, reproducible states as evidenced by natural experiments that measure statistical distributions of stress from failure, strain energy, and scale-dependent rock strength. Knowing the mathematical form, and even establishing existence of the distributions has proven to be a powerful tool for tracking changes, characterizing systems and explaining rupture behavior. If the primary role of earthquakes in the system is to redistribute stress, then self-organization is a central feature of the Earth’s crust.

Earthquakes occur when stress on a fault overcomes the strength. Stress is increased over time as plates move and strain the elastic crust. Strength is determined by the frictional stress required for failure. Ultimately, stress overcomes strength and an earthquake occurs.

This simple paradigm suggests a degree of predictability in the earthquake cycle. We should be able to measure fault loading through geodetic methods. We should also be able to measure the strength of rocks by measuring friction through a combination of laboratory and field techniques (1–3). We should, in principle, be able to use these measurements together to predict earthquakes.

In practice, things are much more complicated. Both loading rate and strength can vary in time and space. Loading rate can vary due to regional and distant earthquakes creating interactions between faults. Transient creep at the base or edges of faults and injection of fluids can further disturb the loading curve. Strength also varies over time and space. Faults can heal frictionally, be flooded with fluids, experience chemical reactions or damage, all of which can change their strength. At the high speeds of earthquakes, friction is lower and more variable than at the low speeds of plate motion (3–5). As a result, the earthquake cycle is far from predictable.

This variability of stress and strength explains, to some extent, our failure to predict earthquakes. Even for some of the best-observed and understood situations, our forecasts are far from perfect. For instance, in every earthquake the slip on a fault distorts the surrounding crust and changes the stresses on nearby faults. This static stress change is commonly quantified as the Coulomb Failure stress change, which is the combination of normal and shear stresses resolved on a fault and is a good predictor of aftershock occurrence in some cases (6–8). However, the predictions are limited. Within the positive stress areas, earthquakes are commonly concentrated in patches that do not coincide with the maximum stress. This mismatch can be attributed to the prestress heterogeneity and seen as an unavoidable manifestation of the inherent variability in the Earth. For aftershock forecasts, this level of predictability may be acceptable.

The issue is more serious when we think about seismicity induced by human activities such as the injection of fluid at depth. In this case, there is a crucial need to control the extent of the earthquake-producing region. For instance, in Fairview, Oklahoma in 2016, a ~15 km-radius injection field induced an earthquake ~30 km from the center of the field (9). Why was this earthquake triggered at such a great distance, and why in this spot? The first part of the question can be addressed by thinking about the elastic stresses that accompany fluid injection. For a finite source, like a large injection field, the elastic stresses extend to distances comparable to the source dimension, which in this case is the diameter of the field, i.e., ~30 km. However, that analysis does not explain why this place and not anywhere else at that radius was triggered. Like aftershocks, the anomaly is attributed to heterogeneity and Fairview just happened to be on a fault very close to failure. That is likely correct, but an unsatisfactory answer from a practical viewpoint.

**Fig. 1.**
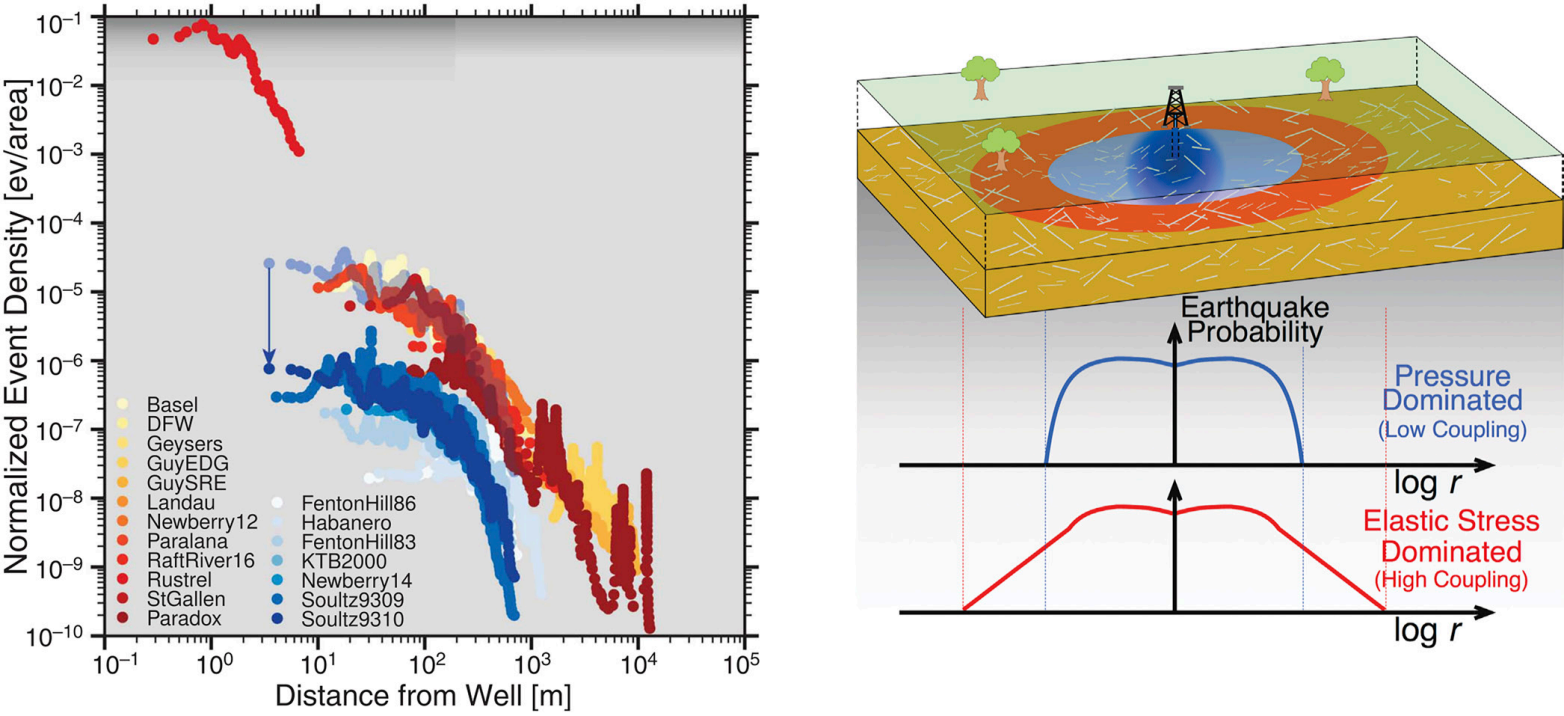
Density of earthquakes as a function of distance from injectors. Observations on the *Left*. Red/orange sites show gradual spatial decay; Blue have a more compact decay. The groups of data overlap as shown by the translucent example. Blue is offset as shown by the arrow for visual separation. With the exception of Basel, the gradual decays are all shallow injection and all of the blue are injected into the basement. Schematic on *Right* illustrates the difference between the deep (blue) and shallow (red) coupling as inferred from the observations (figures reproduced from ref. 10, AAAS).

Goebel and Brodsky take a different approach and studies how likely a far-away earthquake is to occur by measuring the density of earthquakes as a function of the distance from an isolated injection well (Fig. 1; ref. 10). The data show consistent, well-defined distributions that fall into two classes based on the type of injection. Shallow injections into sedimentary units create seismicity densities that decay as a power law extending to large distances from the well, while deep injections create a compact cloud. From a hazard point of view, we now have a tool to assess the probability of earthquakes at a given distance from an injector. Moreover, there is a mechanistic implication that the shallow injectors are triggering seismicity by a different mechanism than the deep ones. Goebel and Brodsky inferred that the shallow injectors trigger seismicity primarily through poroelastic stresses, which extend to great distances when the injection is in soft rocks, and the deep injections trigger seismicity primarily through direct pore pressure effects in the stiff basement rocks (10).

The success of the Goebel and Brodsky approach implies that the probability distribution of stress required to trigger earthquakes in the crust may be well-defined and relatively consistent from region to region. This observation suggests a path forward for studying the variability of stresses in the crust. What we should do is to concentrate on using populations of observations to measure statistical descriptions of stress and related quantities. Then, if we find that the distributions are consistent across regions, we can use these distributions, rather than individual cases, as interpretative tools.

More fundamentally, if such stationary distributions do exist, then the question is why. How do earthquakes organize stress, and related quantities such as energy and strength, into reproducible, well-defined, stationary distributions. What does the existence of these distributions tell us about the earthquake system? This paper focuses on addressing this question. We first review a few illustrative studies that observationally capture the distribution of stress or the related quantities of energy and strength. We then show how the form, or even just existence, of these distributions can be used to track changes in systems, compare systems and explain puzzling behavior. Ultimately, we speculate on why specific, well-defined distributions are common in the Earth and arrive at a scenario where the primary function of earthquakes is to redistribute stress so that the stationary states emerge.

## How Can We Observe the Distributions?

### Stress.

One of the best observational routes into measuring in situ stress is to use known forcings and measure the number of resulting earthquakes. Dynamic triggering, where seismic waves from one earthquake trigger another, has been a particularly useful probe. Since the seismic waves can be measured and well-described by elastic theory, the corresponding applied dynamic stresses are known. Thus, the triggering stresses are known, at least under circumstances where the seismic waves are the only plausible trigger. In the nearfield, one earthquake might be triggering another through a combination of static stress, poroelastic and creep-assisted processes. The situation is much simpler at great distances, since the dynamic stresses decay with distance much more slowly than the other processes. Thus, at great distances, the natural experiment is very well-controlled. Even in the near-field, if there is a clear trend of earthquake rate with peak wave amplitude, dynamic triggering can be inferred (11).

Interevent time distributions can be used to measure the dynamically triggered rate change (12). The idea is to examine the time to the first earthquake in a region following the arrival of seismic waves, relative to the time of the prior earthquake in the region. The interevent times are combined for many different, distant earthquakes that have similar local amplitude to develop a combined view of the effect of seismic wave amplitude on the region. If earthquakes after the seismic wave arrivals are biased to occur sooner than expected based on the prior seismicity, triggering is inferred and a relative rate change is calculated for that wave amplitude.

**Fig. 2.**
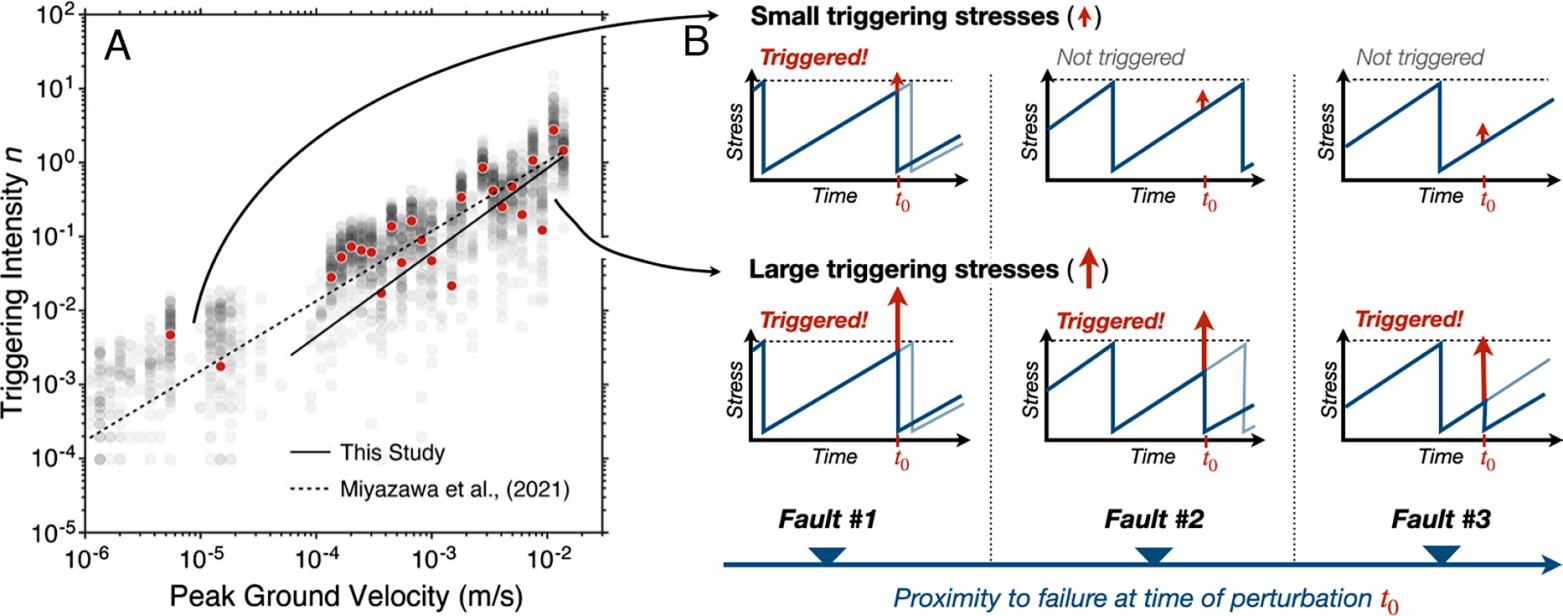
Triggering rates in Southern California from two different studies of dynamic triggering and interpretation in terms of the required stress for failure. (*A*) Triggering intensity is a proxy for change in earthquake rate relative to background rate and is measured from the distribution of interevent times. Peak Ground Velocity is proportional to the dynamic stress applied by the triggering seismic waves. (*B*) Cartoons of typical earthquake cycles and the effect of the potentially triggering stress. *Top*, a small applied stress from a small peak ground velocity can trigger a small number of faults near failure. These small stresses therefore result in small triggering intensities. *Bottom*, a large applied stress from a large peak ground velocity can trigger the fault both far and close to failure. The resulting triggering intensity is therefore large (panel *A* adapted from ref. 14, which is licensed under CC BY 4.0).

The initial observations of rate changes based on interevent times suggested that the triggered seismicity rate in Southern California from 1981 to 2008 was proportional to the applied strain ε to the power of 0.43 (12). Interevent time observations in Fig. 2 using an entirely independent dataset from 2008 to 2017 showed a nearly linear dependence of rate change on strain (ε^0.944^) when measured from the timing of the first event after a triggering strain compared to background distributions (13). The same study also used an alternative measurement strategy based on measuring the timing of the prolonged sequence that results in triggering productivity proportional to ε^1.1^. A more recent measurement based on interevent times measured directly from detections in the seismograms from 2015 to 2021 (as opposed to preassembled catalogs of events) has a fit of ε^1.14+/−0.24^, which is again consistent with a linear relationship between triggered earthquake rates and strain (14). The consistency of the most recent measurements suggests that the earlier ε^0.43^ measurement of ref. 12 was biased by the sparse earthquake catalog available at the time. The early study had insufficient data to consistently have a local earthquake in every location studied before and after every triggering strain and therefore relied on catalog simulation to correct for the sparse data and the nonstationarity in the triggering times. The later studies had sufficient data to measure interevent times in a small time window around each triggering time and did not require any such simulations. Therefore, the two later studies are likely the most reliable and in Southern California triggering rate depends linearly on the amplitude (strain) of the triggering waves as shown in Fig. 2.

The data can now be interpreted as a measure of the cumulative distribution function of stress from failure (Fig. 2). A small perturbation triggers the faults extremely close to failure (15). These faults are relatively rare, and thus the triggered seismicity rate is a small increase relative to the background. A larger perturbation can trigger many more faults, including both those relatively far from failure as well as those that were quite close to failure (Fig. 2*B*). Thus, the seismicity rate for a large perturbation is an integrated measure of the number of faults within that stress of failure.

For Southern California, the nearly linear cumulative distribution function is indicative of a uniform distribution of stresses required to trigger faults. On average, there are just as many faults 1 kPa from failure as 10 kPa. Faults seem to be distributed evenly over their loading cycles. This remarkable and repeatable observation shows a surprising degree of self-organization in one of the world’s best-monitored fault systems.

### Energy.

The stress to failure determines where and when earthquakes begin. How big the earthquakes get is governed by the available strain energy. From a fracture mechanics point of view, the strain energy delivery rate *G* governs crack growth (16, 17). In the Earth, the patchwork of prior ruptures and fault loading creates a complex topology of strain energy that governs the available energy for growth. Observing any aspect of the strain energy distribution is a major challenge in earthquake science.

**Fig. 3.**
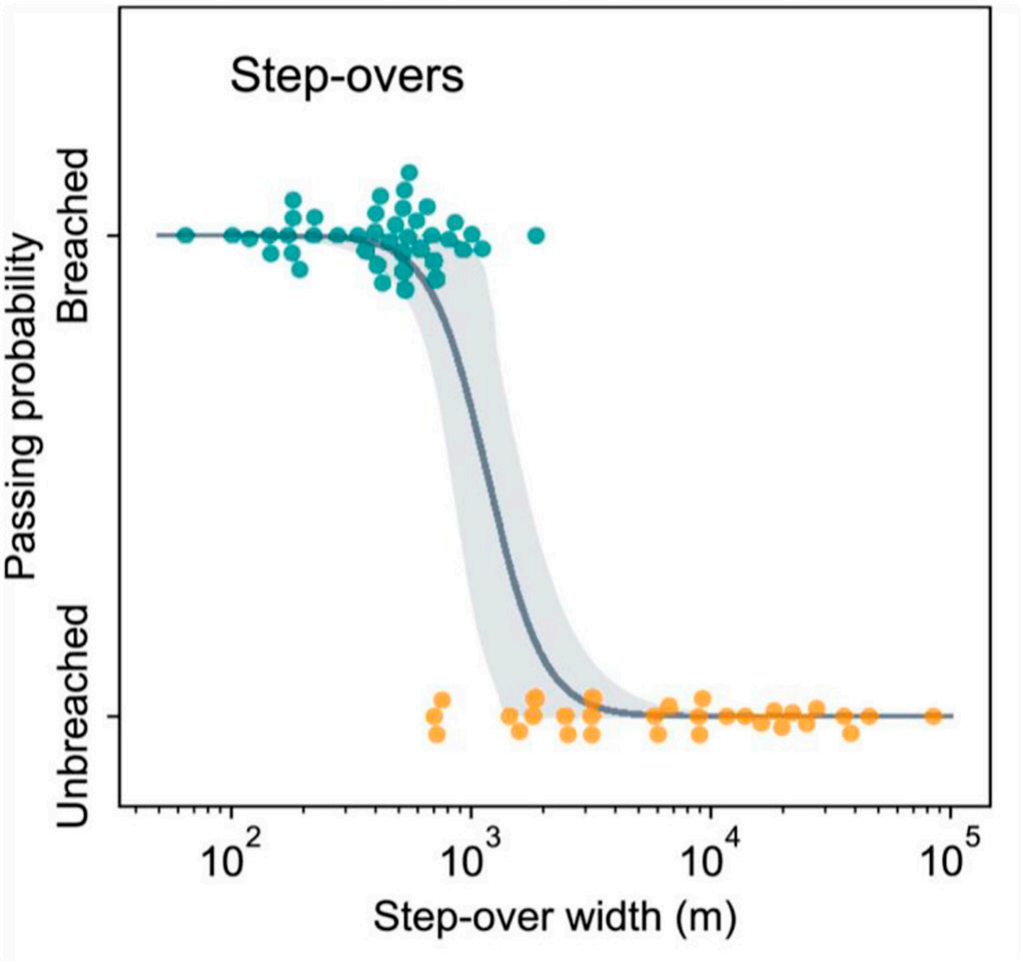
Observations of breached and unbreached step-overs as a function of step-over width in mapped surface ruptures of strike-slip earthquakes M5 and above. The line is the best-fit logistic function and the shaded area is the 95% CI (figure reprinted from ref. 18, which is licensed under CC BY 4.0).

Measuring the likelihood of a rupture propagating over a known barrier provides statistical information about the energy availability. Rodriguez Padilla and colleagues measured the probability of mapped strike-slip ruptures propagating through step-overs (Fig. 3) and found that step-overs ~1 km wide could stop ruptures (18). Nearly all stepovers less than this width were breached by ruptures, while nearly all greater than this width formed a barrier. Interestingly, this threshold was consistent regardless of the distance the rupture traveled before encountering the step-over.

This step-over width corresponds to a threshold of strain energy delivery rate *G*. The more energy delivered to the stepover from the incoming rupture, the higher the stress concentration at the tip and greater the distance at which those stresses are significant. Whether or not a given offset segment is ruptured depends also on the prestress on that segment. Surprisingly, the data show that for a given offset, the prestress does not seem to be producing different behavior. Simply the distance to the rupture tip is the controlling quantity.

The exact value of *G* corresponding to the threshold offset distance depends on the fracture mechanics model, and there is no simple analytic solution for this case. However, the consistency of *G* is still interpretable. The whole population studied had the same critical barrier size corresponding to a particular value of *G,* that we will call *G_s_*, that stops the earthquakes. Since all of these earthquakes are stopped by the same critical *G_s_*, the ruptures had the same *G*, when arriving at the barrier. This particular study involves strike-slip earthquakes with surface rupture, which are necessarily moderate to large earthquakes and often saturated the seismogenic zone. It seems that these ruptures achieved a steady-state energy delivery rate governed by the width of the seismogenic zone. Given the heterogeneity of prestress and material properties in the Earth, such a steady state is not a foregone conclusion. We might expect the strain energy to be variable in the crust due to the complex history (19). Surprisingly, the available energy *G* is constant here and the available energy is sufficiently well-distributed that a steady-state is possible. Again, the Earth is surprisingly well-organized.

### Strength.

Strength is also variable in the crust. Most of our current knowledge of rock strength comes from laboratory experiments on centimeter-scale samples. Earthquakes slip on faults that can be 1000s of kilometers long. Extrapolating strength to these scales requires observations that also exist over the same range of scales.

One such observation is fault roughness. Fault surface topography can be measured at the laboratory scale through profilometry, at the field scale by ground-based LiDAR or structure from motion, and even at a larger scale using 3D active source seismic data of subducting plate surface (20–23). These measurements have shown that the roughness of a fault surface follows well-defined distributions with the mean asperity height increasing as a power law function of the scale of observation. Most fault measurements have *H*~*L*^0.6^ in the slip direction and *H*~*L*^0.8^ in the slip perpendicular direction. The fault roughness both records the effects of prior slip and sets the conditions for the next slip event.

The consistent, reproducible distribution reflects the interplay between the geometry and the strength of the fault material (21, 24, 25). As two rough surfaces slide past each other, bumps (asperities) collide and must deform to accommodate slip. For an asperity of average height *H* and length *L*, the strain needed to push an asperity past another one is that required to fully flatten one of the asperities. The flattening involves a surface displacement *H* accommodated over the width *L* and so the requisite shear strain is of order *H*/*L*. If the surface is smooth enough, the shear strain remains below the strength of the material. Then, the deformation is elastic and asperities can squeeze past each other unscathed. However, when an asperity is too tall or too thin, the required strain exceeds the material strength. In that case, the asperity yields inelastically. The plastic or brittle deformation erases this asperity from the rock record.

Thus, the preserved fault roughness reflects the strength of the material, at a given scale. Like many materials, rocks have scale-dependent strength: large rocks are weaker than small ones (26). As a result, faults are rougher at the small scale than at the large ones with (*H*/*L*)_small_ > (*H*/*L*)_large_. The observed scaling laws of fault roughness are the expression of the scale-dependent rock strength.

**Fig. 4.**
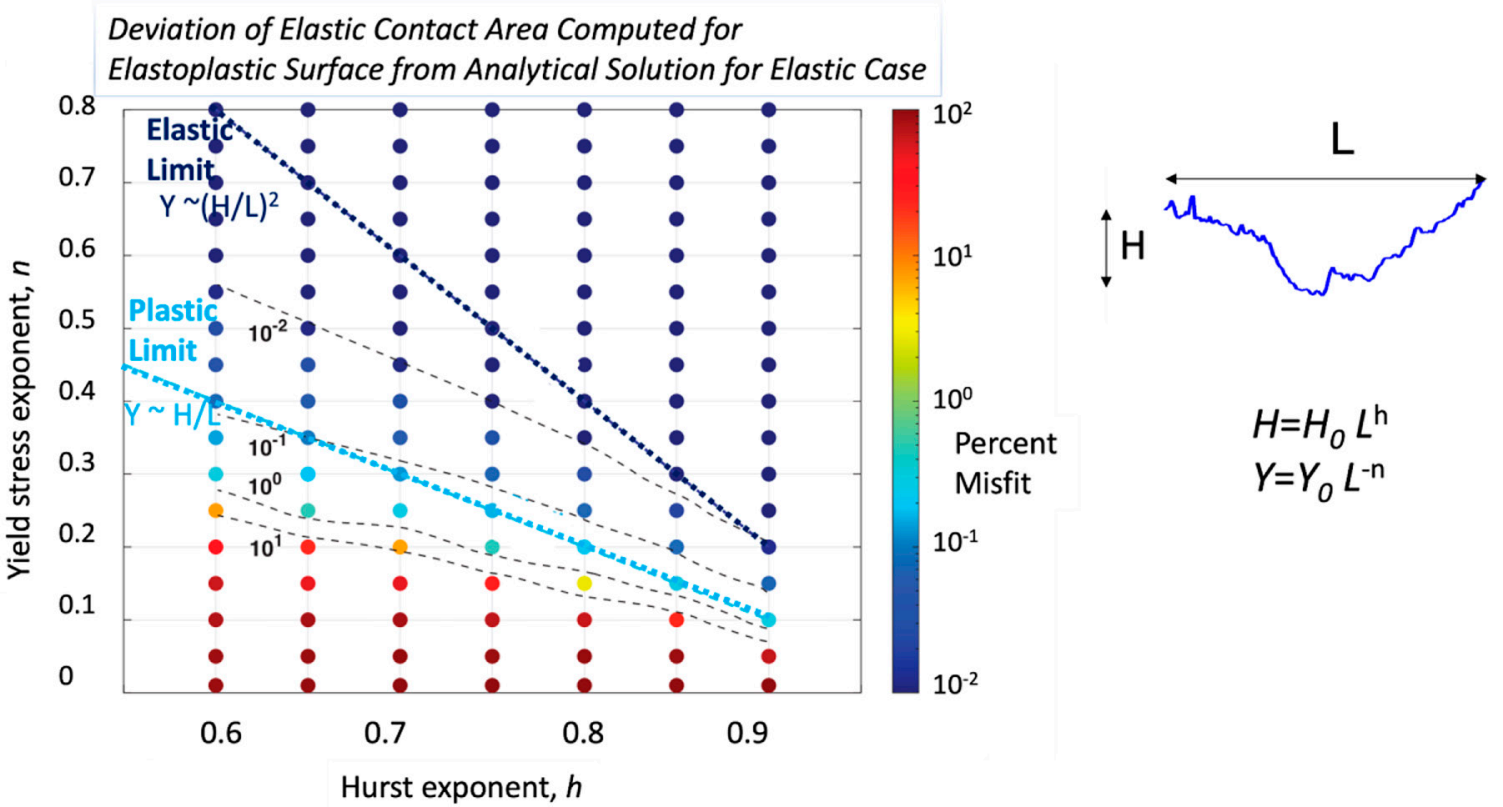
Regimes of preserved or deformed roughness on an elastoplastic surface. Contours and colorscale show deviation of the elastic contact area from a purely elastic solution for an elastoplastic self-affine surface with scale-dependent yield strength. The roughness average asperity height *H* scales with lengthscale *L* as *H*~*L^h^* and the yield stress Y scales as *L^-n^*. The more the deviation, the more plasticity affects the solution, and the more irreversible deformation is required. No deviation exists beyond the elastic limit, and only moderate deviation (~10%) above the plastic limit. Therefore, surface roughness above the plastic limit is likely to be substantially preserved (figure reprinted with permission from ref. 27, Copyright 2025 by the American Physical Society).

This scaling argument is supported by a more rigorous analysis of the elastoplastic case (27) that builds on the statistical contact mechanics theory of Persson (28–30). The formal treatment shows that the relationship between surface roughness and scale-dependent strength is governed by two limits. One is a plastic limit which is the same as that derived by the scaling argument above, i.e., the yield stress *Y*(*L*) is proportional to *H*/*L*. If faults are rougher than what the yield stress prescribes, nonrecoverable deformation dominates and smooths them (Fig. 4). The theory also reveals another, distinct elastic limit that defines the condition where all deformation can be accommodated elastically and all roughness will survive. The elastic limit occurs when strength scales (*H*/*L*)^2^ as shown in Fig. 4. Numerical solutions show that the inelastic deformation of surfaces between the elastic and plastic limits is small and thus real surfaces likely have roughness that sits between the elastic and plastic limits (27).

One more time, we are seeing that faults evolve to a consistent distribution of an observable quantity. Here the quantity is fault geometry, which is a proxy for scale-dependent strength. In this case, there is a physical rationale and partial model for the consistency of the roughness scaling. The key ingredient of the model is the intrinsically stochastic approach to the equations (29). In the future, we hope that a similar approach could bear fruit for the other distributions discussed in this paper.

## Applications

We have now established that consistently observable distributions of stress, energy, and strength exist. Before examining their origins in greater depth, we pause to apply the information we already have. In a first application, we will utilize the success in measuring the stress from failure from dynamic triggering to measure how those distributions change in response to a major earthquake. The second application will show how increasing complexity of fault systems results in increasing degrees of desynchronization, with an end-member being the uniform distribution of Southern California. In this case, the distributions of stress states are a useful discriminant to identify and categorize tectonic systems. As a final application, we will move to laboratory experiments and show how simply knowing about the existence of stationary distributions allows us to understand the stress drop and reloading during the earthquake cycle.

### Application 1: Using Distributions to Measure Changes.

**Fig. 5.**
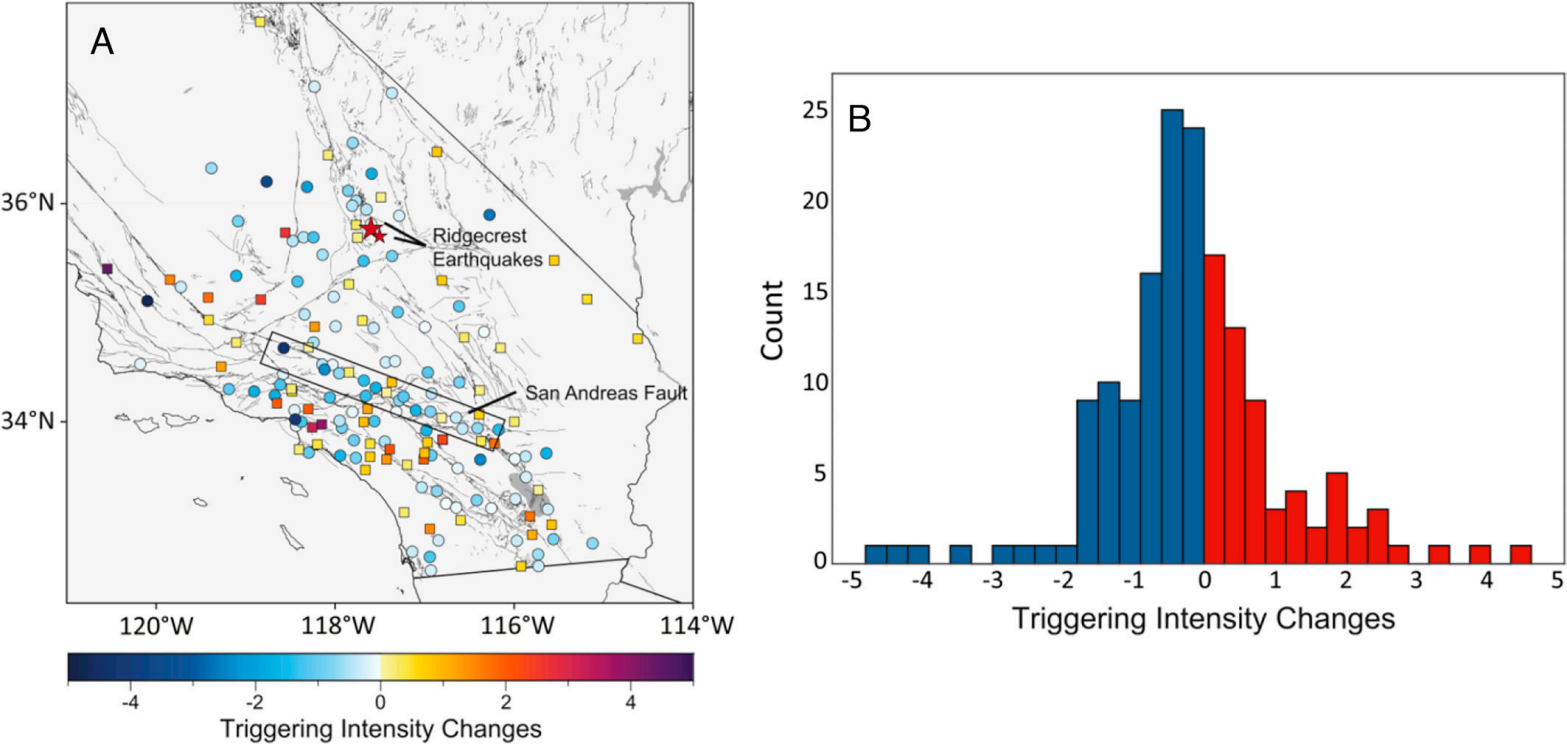
Triggerability changes in Southern California following M7.1 2019 Ridgecrest from 1 mo after the event through 2021. Triggerability index is a measure of the triggering intensity *n* at a reference value of ground motion, which allows for comparison of the relative triggerability between stations. (*A*) Map of triggering intensity at each station. (*B*) Histogram of stations showing that more stations had their triggerability reduced than increased (figure reprinted from ref. 14, which is licensed under CC BY 4.0).

The single-station based measures of triggerability developed in Ref. (14) provide a new, highly sensitive method to measure changes in the crust. The study compared the period from 2015 until the 2019 M7.1 Ridgecrest earthquake to that following Ridgecrest until 2021, omitting the month immediately after Ridgecrest as it is dominated by the aftershocks of the mainshock. Surprisingly, many stations in the region had reduced triggerability following Ridgecrest (Fig. 5). Stations along the San Andreas were particularly prone to the reduction of triggerability. Perhaps the Ridgecrest event triggered all faults near failure immediately, and in the months and years following, few near-failure faults remained to be triggered.

The study illustrates the power of using distributions, rather than individual events, to study changes in the crust and reveal potentially important patterns about the proximity to failure.

### Application 2: Using Distributions to Compare Regions.

A second application of distributions is to tease apart key distinctions between regions. Consider one of the most spectacular datasets produced in recent years. Tectonic tremor accompanying slow slip episodes was discovered in the early 21st century due to the increased density and decreased noise level of networks (31, 32). Episodic tremor and slow slip is particularly prevalent in subduction zones. The catalogs of tremor events track the activity of slow slip in high resolution, revealing patterns of unusual regularity for a geologic phenomenon (33).

**Fig. 6.**
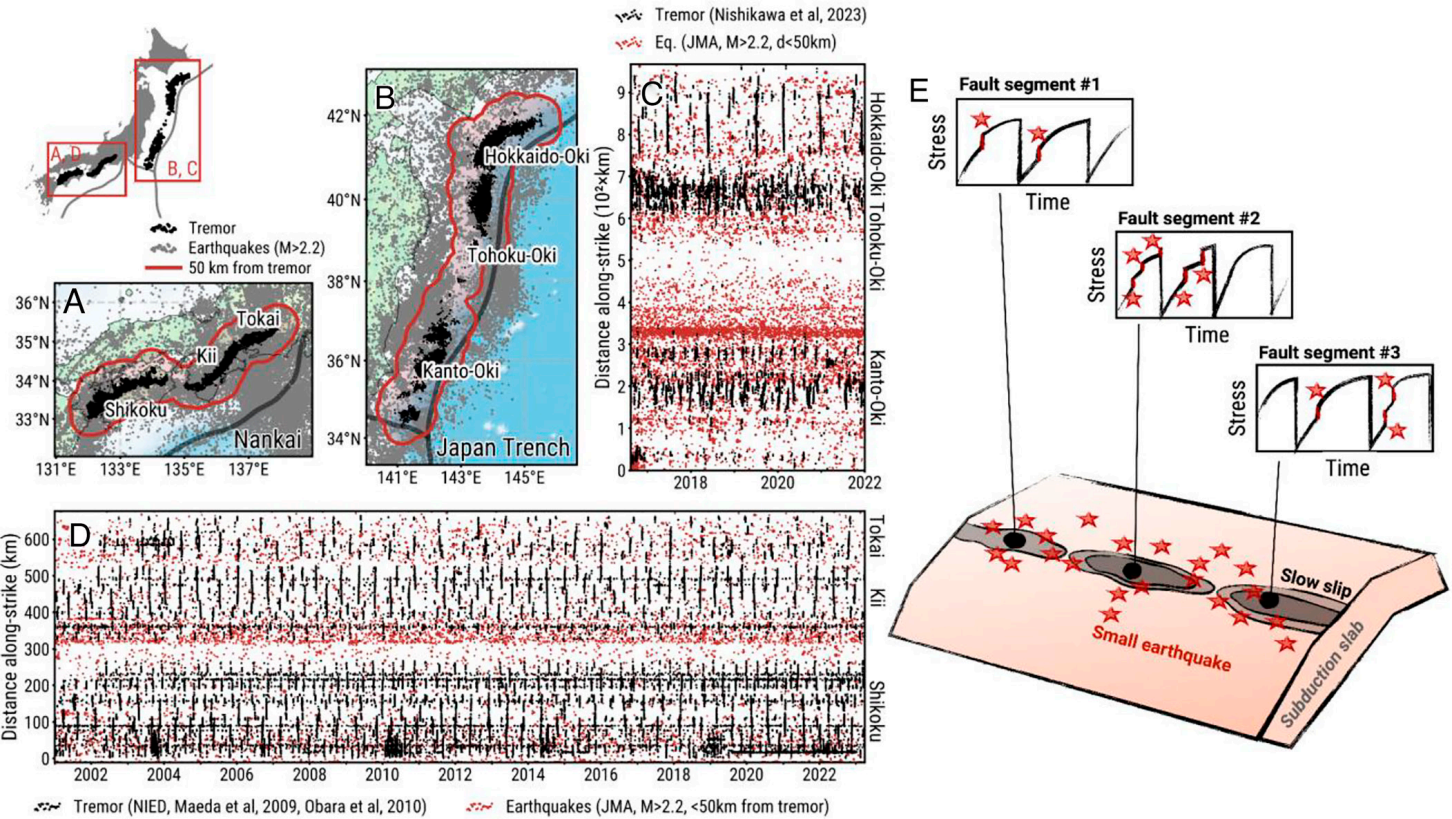
Synchronization of tremor in Japan and cartoon diagramming synchronization. Maps of tremor (black) and earthquakes (gray) in (*A*) Nankai and (*B*) the Japan trench. Earthquakes are from the Japan Meteorological Agency (JMA) catalog with M > 2.2. The red contour indicates the area in which earthquakes are less than 50 km away from the tremor. Time series of tremor and regional earthquake activity within 50 km of a tremor event in (*C*) the Japan trench and (*D*) Nankai. Most of the regional earthquakes are not on the plate boundary but rather occur in the crust of the upper plate or within the slab (figure reprinted from ref. 34, which is licensed under CC BY-NC 4.0). (*E*) Each small earthquake locally perturbs the stress evolution on the fault and thus prevents neighboring segments from synchronizing.

Farge and Brodsky suggested that slow slip synchronizes on the fault along strike due to a common feature of nonlinear oscillators (34). Like croaking frogs or fireflies (35), fault cycles tend to synchronize with their neighbors in the absence of any perturbation (36). However, perturbations such as the stresses from regional earthquakes locally disturb some of the fault patches and desynchronize the system, much like the sounds of an intruding hiker desynchronizes the songs of frogs in a pond. Farge and Brodsky (34) indeed observe that regions of high intraslab or crustal seismicity have less synchronized tremor activity than seismically quiet regions (Fig. 6).

**Fig. 7.**
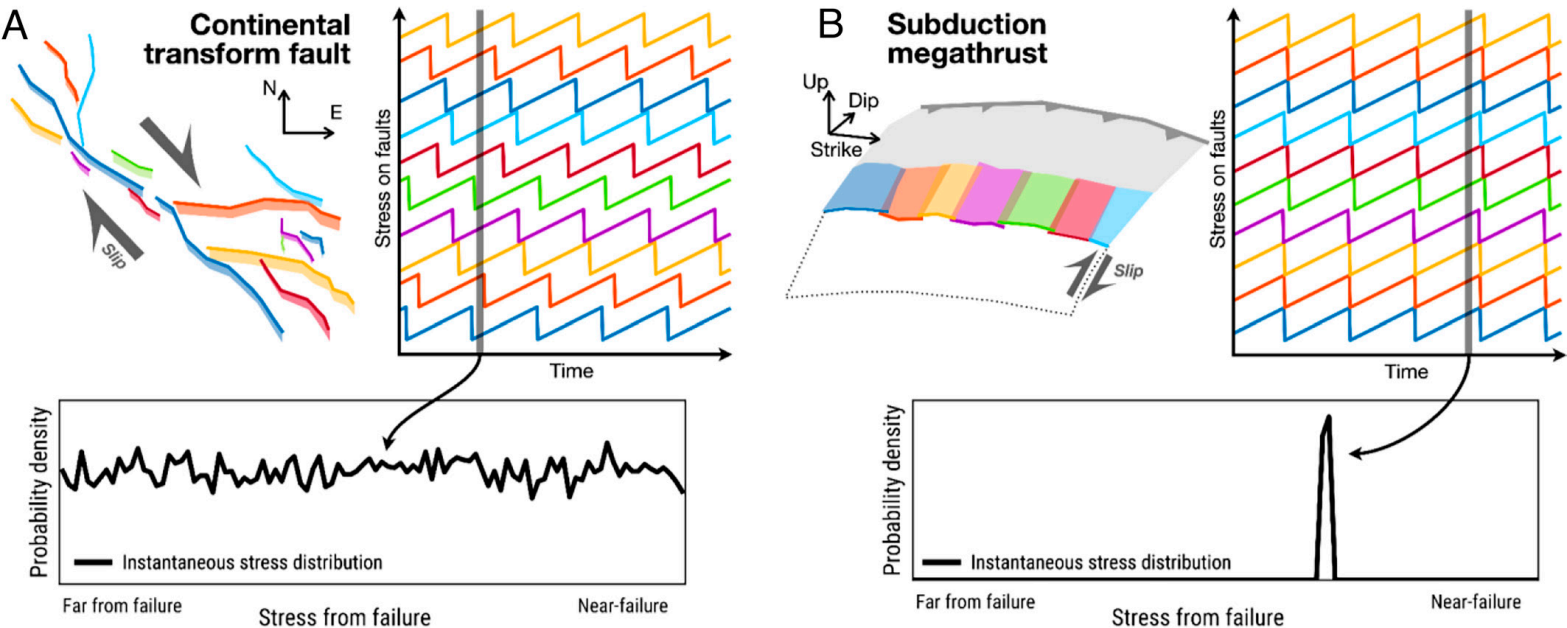
Schematic cartoon of the distinction between continental and subduction settings. (*A*) A continental fault system is complex with desynchronized cycles. Each colored fault corresponds to a time series of stress. For illustrative purposes, the recurrence intervals are all identical in this cartoon. The *Bottom* panel shows a schematic of the probability distribution of the stress from failure at an instant in time, which is approximately a uniform distribution. (*B*) The same schematic for a subduction setting, where the fault segments are aligned along the interface of the plates, and the cycles are synchronized. The resulting distribution of stress from failure at any given instant is a delta function.

In the present discussion, the synchronization observation is significant because it implies a fundamentally different stress distribution in these subduction zones than in the continental system of Southern California. In Southern California, the dynamic triggering studies show a uniform distribution of stress from failure, which suggests earthquake faults exist at a range of stages of their cycle. In contrast, in the tremor situation, the synchronization means that all the tremorgenic faults in a region are at the same phase of their cycle. The stress from failure is the same for all of them, i.e., the distribution of the stress from is a delta function (Fig. 7). We speculate that the difference in distributions is a fundamental characteristic of the subduction versus crustal fault system. The subduction system is simpler, the faults defining the megathrust are aligned along a clearly defined primary, mature plate boundary. Therefore it has a simpler stress distribution. On the other hand, the complex, anastomosing fault system of the crustal plate boundary produces a more distributed stress distribution (14). Of course, the observation at present is a comparison between earthquake and slow slip activity recorded by tremor, so one could also attribute the distinction to the difference in rupture style rather than the fault system. Other factors, like the form of the loading curve or diversity of recurrence intervals can contribute to synchronization versus desynchronization (35) and vary place to place. The key point is that viewing the observations through the lens of stress distribution provides a way to distinguish and categorize faulted regions in the Earth.

### Application 3: Using Distributions to Explain Behavior.

The final application of the stress distributions is to explore how the variability of stress can create behavior that is otherwise enigmatic and cannot be explained by homogeneous systems. We examine a laboratory experiment of faulting to illustrate this application.

**Fig. 8.**
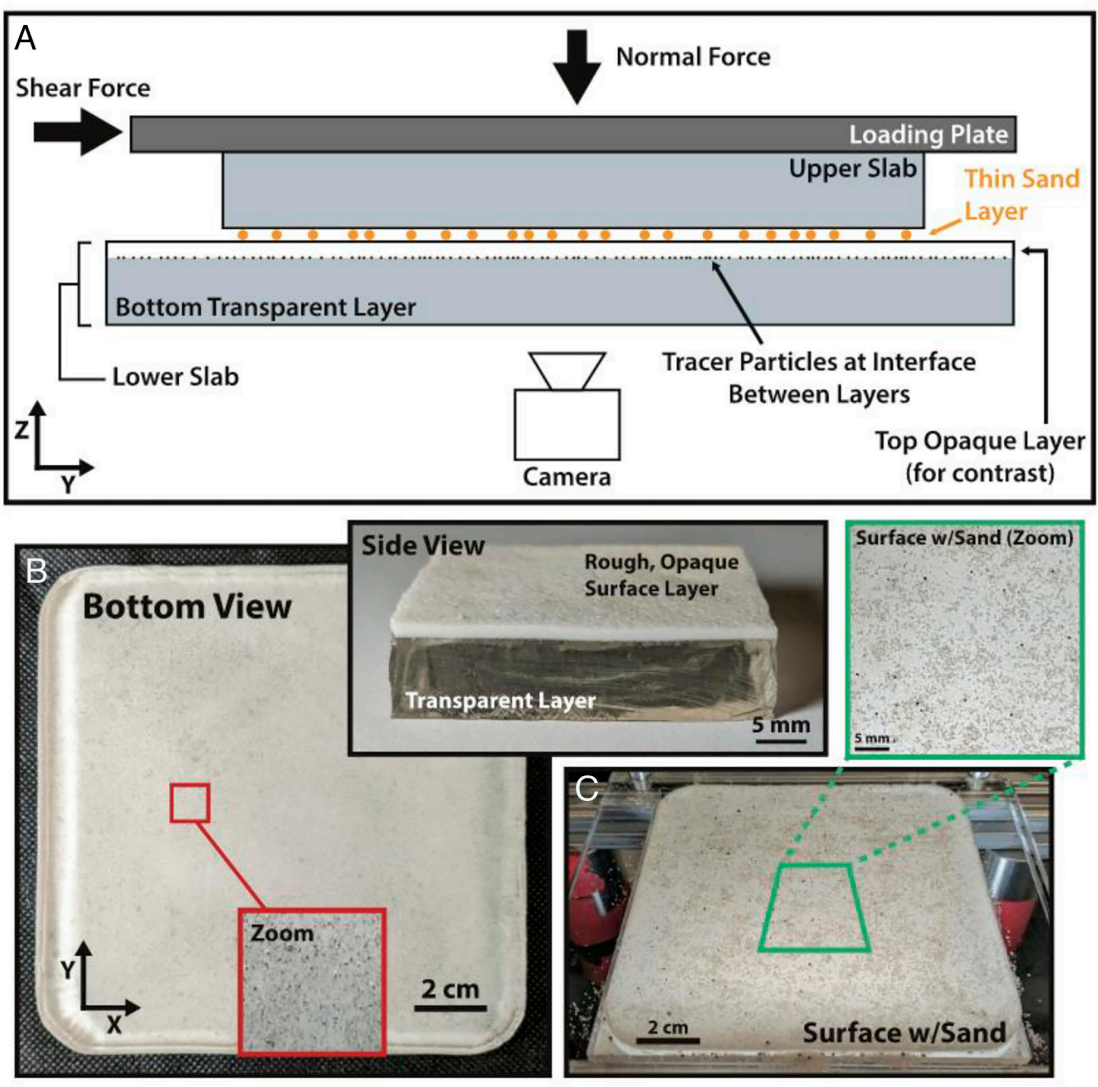
Experimental configuration. (*A*) Schematic cross-section of the experiment. Bulk normal and shear loading are both controlled and measured, and local strains are measured through the displacement of tracer particles embedded in the lower slab ∼1 mm below the frictional interface. A thin sand layer is placed between the slabs to allow nucleation of events everywhere on the sample (*B*) (Side view) Cross section of sample showing thick transparent layer and thin opaque layer of the same composition. The top of the opaque layer, which is cast onto sandpaper, is the fault surface. Tracer particles are embedded between the two layers and viewed through the transparent layer. (Bottom view) Sample viewed from below with inset red box showing a zoomed in region displaying how tracer particles are viewed by the camera. (*C*) The interfacial sand layer is well‐distributed across the interface and is approximately one grain layer thick (figure and caption reprinted from ref. 38, which is licensed under CC BY 4.0).

Steinhardt et al. (37) presented a scale model built of transparent rubber (PDMS) that allowed sequences of slip events to be simulated (Fig. 8). The primary data collected are a movie of these slip events as they rupture different parts of the surface over time. Importantly, because the material is soft, most slip events are entirely confined within the laboratory fault. The surface is seeded with a thin layer of sand that allows slip to nucleate anywhere on the fault. This results in slip events redistributing strain energy to the edges of the slip patches and, after a few earthquake cycles, a heterogeneous stress distribution emerges. This naturally created heterogeneity then influences the qualitative and quantitative behavior of the earthquake parameters commonly observed by seismologists.

**Fig. 9.**
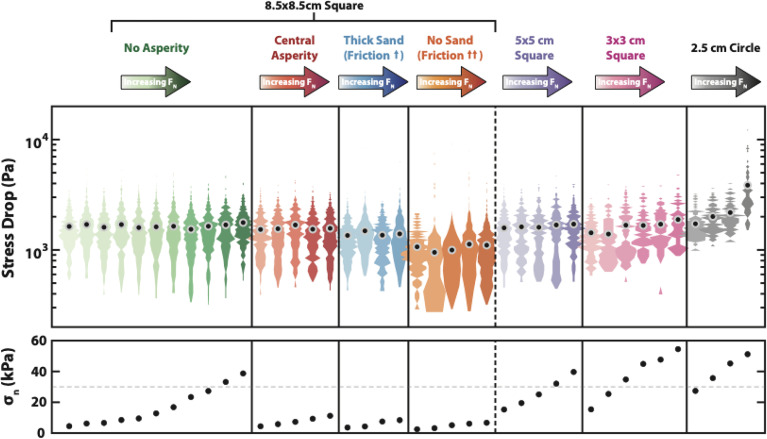
Laboratory measurements of stress drop on slip events on a transparent rubber interface. Colors denote each experimental regime. For each experimental region, a suite of experiments with increasing normal stress was performed (*Bottom* panel). The width of each shape of stress drops corresponds to the probability density function for those conditions. Stress drop only covaries with normal stress for the small samples (*Right*-hand side) (figure reprinted from ref. 37, which is licensed under CC BY 4.0).

For instance, the stress drop of the individual slip events in the experiment is independent of the normal stress on the fault (Fig. 9). Here the definition of stress drop matches common seismological practice, i.e., stress drop ~ *M_0_*/*L*^3,^ where *M_0_* is seismic moment and *L* is the rupture length. The laboratory study produces the same spread of stress drops for a wide variety of experimental conditions, regardless of the normal stress applied to the fault analog. This behavior mimics the largely depth-independent stress drops observed in the Earth (39), but is at odds with the common understanding of stress drop as proportional to the change in friction stress on the fault (40, 41). Regardless of the details, frictional force is proportional to normal force and therefore, stress drops are expected to be proportional to normal forces in a simple model. The fact that stress drop does not change significantly with depth, combined with this conceptual understanding of friction, might demand high pore pressure at depth in the crust (42). Intriguingly, the laboratory experiment produces the same behavior without any fluid involvement.

The normal stress-independence of stress drop seen in the lab vanishes for experiments with smaller samples. The normal stress-independence seems to require stress heterogeneity within the sample due to the history of prior ruptures that create stress concentrations on the fault. This phenomenon can only be experimentally reproduced on analog faults that are less stiff than rocks, where ruptures are confined rather than extending to the free surface of the sample. Steinhardt et al. (37) interpret the data as evidence of the importance of an emergent, naturally created stress distribution. This view is similar to prior work that emphasizes the role of small earthquakes in redistributing the stress field (43, 44). At a macroscopic scale, this distribution may be stable enough to create a consistent stress distribution regardless of normal stress. If the normal stress changes, the stress organization might change so that the average stress change in a slip event is constant.

Another effect of the stress heterogeneity is to create locking events. The laboratory fault has a complete sequence of locking events in addition to slip events (38). The locking events are predictive of future locations and time of slip. Unlike common fault models, locking is spatially heterogeneous and occurs for only part of the cycle and part of the fault. The locking is influenced by the prior stress distribution from the prior history. They have a power law magnitude distribution, much like the slip events. These locking events would not occur without a particular distribution of stress. As before, recognizing that stress distributions can self-organize is the key to understanding why the fault slips as it does.

## Why Do Well-Defined Distributions Exist?

The observational evidence for well-organized distributions of stress, energy, and strength in the Earth raises an obvious question. Why do such distributions exist?

The explanation may lie in the underlying mechanics of friction. Heterogeneity is an intrinsic and required part of friction. The resistance to sliding is not proportional to the normal force on a perfectly smooth surface. Friction proportional to the normal force requires a microscopically rough surface with only a small proportion of the surface in contact. This real area of contact adjusts in response to the normal force (45–47). The elastic interactions between contacts combine with the intrinsic asperity strength to create a distribution of stresses that in aggregate can support the load. Thus, specific distributions of stress emerge on the surface from the contact mechanics (29). Granular flow friction is analogous to contact mechanics, but in higher dimensions. Some grains carry more of the load than others. Elasticity couples stresses across the system to form structures such as force chains. The resulting distribution of stresses is robust regardless of experimental details (48, 49) and suggests again that self-organization is intrinsic to frictional systems with long-range elastic forces.

Similarly, fault systems are a network that cooperatively accommodates tectonic loading (50). In the farfield, the plates load the network. That load is accommodated through a combination of strands, some of which are highly stressed and others that carry relatively little load. Again, elasticity plays an essential role in connecting the system by providing a long-range force that allows for collective organization in the system: each fault playing its role in bearing a share of the tectonic stress. As a result, variability of stress results. Fault stresses may organize into a consistent, reproducible distribution, much like the frictional asperities and granular systems.

In this scenario, the more complex the fault system, the more broadly distributed the stress to failure. Equivalently, faults are broadly distributed over a large range of stages of the loading cycle. By this logic, continental systems should result in more distributed distributions than simpler, subduction systems which are dominated by the principal slip surface of the megathrust. In dynamical systems, increased disorder is known to frustrate synchronicity in an analogous process (51). To some extent, this analysis is supported by the data discussed above. The synchronicity of tremor in Nankai, Cascadia, and other subduction zones implies synchronized loading cycles and a narrow distribution of stress states (34). In contrast, the dynamic triggering data in Southern California imply a broadly distributed stress state (14) (Fig. 7).

If the stress distributions in the crust evolve to preferred states, the mechanism of evolution must be the earthquakes themselves. Earthquakes redistribute stress. As suggested by the laboratory experiments, the stress states evolve over history. Stress is released in one location and deposited somewhere else in each event. The examples described above seem to suggest that this redistribution leads to a dynamic but consistent distribution of stress in the Earth. Such a scenario implies that the most important energetic function of earthquakes is to redistribute energy, which sets the scene for the well-organized seismic system as observed.

These observations and inferences are reminiscent of self-organized criticality, where a system of faults evolves to a state where a small perturbation can produce an arbitrarily large event. The evidence for self-organized criticality for earthquakes originates in studies of the seismic magnitude–frequency relationship and its tendency to have a slope (b-value) of 1 on a semilog graph (52, 53). The attraction to this function across all observable magnitudes suggests a self-organized system capable of events of all sizes in response to an arbitrarily small trigger. These intriguing interpretations of magnitude distributions are challenging to apply to fault systems with definite architecture and limits to interactions. Self-organized criticality models usually require homogeneous coupling and a large number of degrees of freedom (54). Faults and localization of strain result in a reduction of the number of degrees of freedom and thus a reduction of the tendency of the system to self-organize to criticality. The more mature the fault, the simpler it is (55), with subduction zone megathrusts forming an end-member. Regions controlled by single, simple faults seem to be synchronized, rather than critical. More complex systems will have more degrees of freedom and approach criticality. For instance, the continental region of Southern California geometry achieves sufficiently complex interaction between nonaligned faults to organize into a uniform distribution of loading states in contrast to the simpler, aligned network of the subduction fault.

To further investigate this point, one could measure the state of criticality from the activity and quantitatively compare it to the complexity of the fault network in a region. Aftershock abundance is a strong candidate metric, despite observational limits. The average number of aftershocks produced by a mainshock in a region is known as the branching ratio and is fundamental to characterizing sequence behavior (56). If the branching ratio is greater than 1, aftershock sequences run away endlessly, which is unphysical. If it is less than 1, sequences expire. The critical state with a branching ratio of 1 has self-sustaining, perpetual aftershock sequences. Such a scenario could only happen if the driving energy balances the dissipation. Since the energy added by plate tectonics is negligible on the timescale of an aftershock sequence, for an earthquake system a branching ratio of 1 implies negligible dissipation. Activity is durably self-sustained through lossless redistribution of elastic energy. The direct observation of the branching ratio is difficult because the cascade of triggering extends to earthquake sizes well below our current observational limits. However, estimates can be derived from statistical models such as the Epidemic-Type Aftershock Sequence (ETAS) model (57–59). Southern California is thought to have a branching ratio approaching the theoretical limit (60, 61). If the branching ratio is indeed near 1, that would again imply that the complex fault network has self-organized criticality. In addition, the primary effect of earthquake sequence in this scenario is to redistribute rather than dissipate energy.

**Fig. 10.**
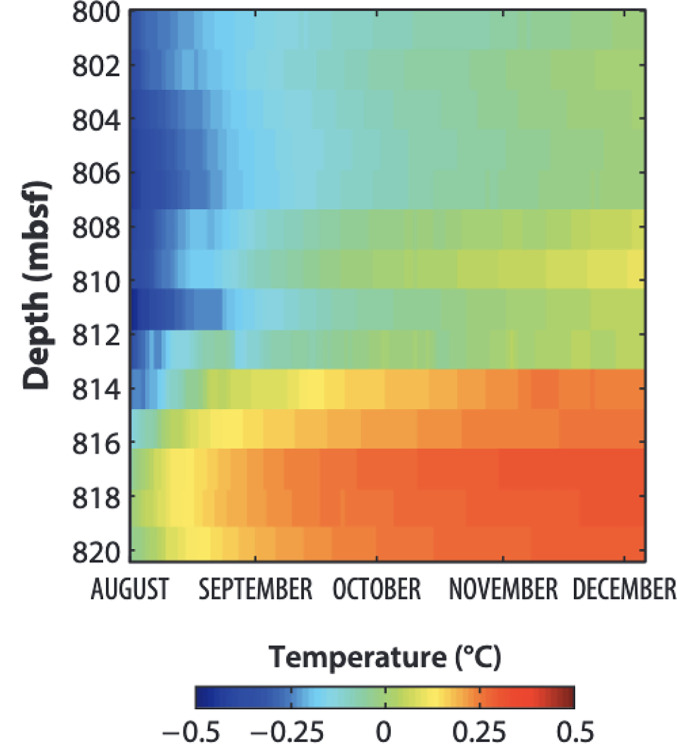
Frictional heat measured in the JFAST borehole. Colors indicate anomalous temperature relative to the background. The fault is inferred to be near 820 m depth below the sea floor. The initial heating of the fault in the data is due to the recovery of the fault from cooling by the cold water that filled the borehole immediately after drilling. The relatively small temperature anomaly resulting from the extreme slip of the M9.0 earthquake implies very low friction during the earthquake (figure modified from ref. 3, AAAS).

Intriguingly, this discussion of statistics and distributions has arrived at a discussion of energy. For the statistics to behave as observed, the energy dissipated in individual earthquakes needs to be small, at least over the timescales that the statistics are measured. There is some evidence that this is indeed the case. The most direct measurement of dissipation on a fault to date comes from the drilling of the M 9.0 2011 Tohoku earthquake (Fig. 10). There, we measured the temperature of the fault after the earthquake and found that the heat energy required that the coefficient of friction was 0.08 (0.05 to 0.15 90% CI), which is significantly below the conventional Byerlee’s Law value of 0.6 (3, 62). The temperature measurements through a borehole under 7 km of water immediately after one the world’s largest earthquakes historic slip are necessarily a single, limited data point. Other lines of evidence pointing to weak faults during slip include laboratory experiments, theoretical models of slip in a granular, fluid-filled, fault and geological evidence of limited heating over large fractions of the fault area (4, 5, 63–67). Earthquakes may be only mildly dissipative and thus sequences are self-sustaining and stress well-organized, at least over short time periods.

In summary, the distributions of stress appear to be self-organized into preferred states, but perhaps not critical ones. This organization is achieved through the earthquake process itself, where stress is redistributed. The redistribution with relatively little dissipation on the timescale of the sequences allows the system to evolve to different attractors (preferred states) in different regions. The preferred attractor may depend in part on the complexity of the fault network. Fault network complexity may also favor criticality.

## A Note About Fluids

Thus far, fluids have been conspicuously absent from this discussion, despite their important role in determining the effective stress on faults. Like elastic systems under load, fault zone hydrogeology also has a potential for self-organization. Earthquakes can increase fault zone permeability by damaging the host rock (68). In addition, seismic waves can increase permeability on distant faults or any fracture-dominated reservoir (69, 70). The permeability increases result in leakage of high pore fluid pressure and thus stabilize the systems against further permeability increases. Similar feedbacks have been invoked to suggest fluid pressure is invariant with depth (42). Observationally, we can observe transient fluid pulses in fault zones after strong shaking (71), as well as rapid healing (72). Perhaps as a result, we observe surprisingly consistent hydraulic diffusivities of ~0.01 to 1 m^2^/s of fault zones in a variety of tectonic settings (73–75). Although this range spans two orders of magnitude, it is much smaller than the range of plausible hydraulic diffusivities in the Earth (76). Talwani and coworkers previously suggested the concept of seismogenic permeability that was controlled by feedbacks to be a restricted range within fault zones (77). Hydraulic diffusivity may be a more natural quantity to be controlled because of the importance of pore pressure diffusion in the system. The potential for a preferred diffusivity governed by earthquake-permeability feedbacks requires that we invest in more long-term hydrogeological observations on faults. Fortunately, new technologies like Distributed Temperature Sensing combined with passive methods may bring such observations within reach (78). The implication may extend well beyond earthquakes. The existence of dynamic permeability enhancement in fractured-rock systems and the accompanying healing over time (70) suggests more generally that fracture-reservoir hydrogeological properties are determined by a dynamic equilibrium rather than simply the geometry and density of the fractures.

## Conclusions

Stress in the Earth is variable. This frustrating fact has held up quantitative understanding of when and where earthquakes occur. Taking a statistical approach is a productive strategy that provides both new observations and new interpretative strategies. Well-defined distributions of stress, energy, and strength are observed. The existence of reproducible distributions provides tools to study otherwise difficult problems such as temporal changes, regional-scale distinctions in behavior and the depth-independence of stress drop. Variability is not a bug. It is a feature emerging from the constant redistribution of stress that is an intrinsic and inevitable part of frictional systems.

The applications explored here demonstrate how fruitful this phenomenological framework could be. Ideally, we would like to use the stationary distributions to predict future behavior of the crust, and its associated earthquakes. Statistical prediction of aftershocks based on empirical relationships is a mature field (58, 79, 80). A major challenge for the future is to connect physical distributions to these empirical relationships. The example of fault strength is particularly inspiring (Fig. 4). There we were able to move beyond scaling arguments by using a theory based on stochastic equations specifically designed to predict distributions (29). Since the observations are of probabilistic distributions, the theory was developed to match. The redistribution of stress by earthquakes is a similar problem that might benefit from a similar theory rooted in its probabilistic observables. The appropriate theory will need to build in the complex or simple structure of faults, and predict their implications for a regions’ degree of synchronicity. Creating such a theory could be the keystone to a more profound understanding of the complexity of tectonics.

Measuring distributions requires vastly more data than measuring and studying individual events. The observations here demonstrate the value of spatially extensive, long-lived instrumental networks. Further advances will require ambitious observational advances, such as instrumenting entire plate boundaries with dense enough sensors to capture the minuscule earthquakes that are constantly redistributing stress. These measurements must be made in context of knowledge of the fault network and constant perturbations from interconnected events. Together, perhaps we can determine how earthquakes organize stress in the crust.

## Data Availability

There are no data underlying this work.

## References

[1] J. Byerlee, Friction of rocks. Pure Appl. Geophys. **116**, 615–626 (1978).

[2] E. M. Anderson, Dynamics of Faulting and Dyke Formation with Applications to Britain (Oliver and Boyd, ed. 2, 1951).

[3] P. M. Fulton , Low coseismic friction on the Tohoku-oki fault determined from temperature measurements. Science **342**, 1214–1217 (2013). 24311684 10.1126/science.1243641

[4] G. Di Toro , Fault lubrication during earthquakes. Nature **471**, 494–498 (2011). 21430777 10.1038/nature09838

[5] K. Ujiie , Low coseismic shear stress on the Tohoku-oki megathrust determined from laboratory experiments. Science **342**, 1211–1214 (2013). 24311683 10.1126/science.1243485

[6] G. King, R. S. Stein, J. Lin, Static stress changes and the triggering of earthquakes. Bull. Seismol. Soc. Am. **84**, 935–953 (1994).

[7] R. S. Stein, The role of stress transfer in earthquake occurrence. Nature **402**, 605–609 (1999).

[8] J. L. Hardebeck , Aftershock forecasting. Annu. Rev. Earth Planet. Sci. **52**, 61–84 (2024).

[9] T. H. W. Goebel, M. Weingarten, X. Chen, J. Haffener, E. E. Brodsky, The 2016 Mw5.1 Fairview, Oklahoma earthquakes: Evidence for long-range poroelastic triggering at >40 km from fluid disposal wells. Earth Planet. Sci. Lett. **472**, 50–61 (2017).

[10] T. H. W. Goebel, E. E. Brodsky, The spatial footprint of injection wells in a global compilation of induced earthquake sequences. Science **361**, 899–904 (2018). 30166486 10.1126/science.aat5449

[11] K. R. Felzer, E. E. Brodsky, Decay of aftershock density with distance indicates triggering by dynamic stress. Nature **441**, 735–738 (2006). 16760974 10.1038/nature04799

[12] N. J. van der Elst, E. E. Brodsky, Connecting near-field and far-field earthquake triggering to dynamic strain. J. Geophys. Res. **115**, 67 (2010).

[13] M. Miyazawa, E. E. Brodsky, H. Guo, Dynamic earthquake triggering in southern California in high resolution: Intensity, time decay, and regional variability. AGU Adv. **2**, e2020AV000309 (2021).

[14] H. Guo, E. E. Brodsky, M. Miyazawa, Triggering intensity changes over time and space as measured by continuous waveforms in Southern California. J. Geophys. Res. Solid Earth **130**, e2024JB030004 (2025).

[15] E. E. Brodsky, N. J. van der Elst, The uses of dynamic earthquake triggering. Annu. Rev. Earth Planet. Sci. **42**, 140228162045009 (2014).

[16] H. Kanamori, E. E. Brodsky, The physics of earthquakes. Phys. Today **54**, 34 (2001).

[17] L. B. Freund, The mechanics of dynamic shear crack propagation. J. Geophys. Res. Solid Earth **84**, 2199–2209 (1979).

[18] A. Rodriguez Padilla , The influence of fault geometrical complexity on surface rupture length. Geophys. Res. Lett. **51**, e2024GL109957 (2024).

[19] D. Morad, S. Gvirtzman, Y. Gil, J. Fineberg, E. E. Brodsky, Under what circumstances is the final size of a laboratory earthquake predictable at the onset of the P-wave? Earth Planet. Sci. Lett. **665**, 119436 (2025).

[20] T. Candela , Roughness of fault surfaces over nine decades of length scales. J. Geophys. Res. **117**, 8409 (2012).

[21] E. E. Brodsky, J. D. Kirkpatrick, T. Candela, Constraints from fault roughness on the scale-dependent strength of rocks. Geology **44**, 19–22 (2016).

[22] J. H. Edwards , Corrugated megathrust revealed offshore from Costa Rica. Nat. Geosci. **11**, 197–202 (2018).

[23] A. Sagy, E. E. Brodsky, G. Axen, Evolution of fault-surface roughness with slip. Geology **35**, 283–286 (2007).

[24] T. Candela, E. E. Brodsky, The minimum scale of grooving on faults. Geology **44**, 603–606 (2016).

[25] R. Aghababaei, E. E. Brodsky, J.-F. Molinari, S. Chandrasekar, How roughness emerges on natural and engineered surfaces. MRS Bull. **47**, 1229–1236 (2022).

[26] C. A. Thom ., Nanoscale roughness of natural fault surfaces controlled by scale‐dependent yield strength. *Geophys. Res. Lett.* **44**, 9299–9307 (2017).

[27] V. Lambert, E. E. Brodsky, Competition between roughness and strength for scale-dependent surfaces. Phys. Rev. E **111**, 065502 (2025). 40745727 10.1103/PhysRevE.111.065502

[28] B. Persson, Theory of rubber friction and contact mechanics. J. Chem. Phys. **115**, 3840 (2001). 10.1063/1.4936558 26671362

[29] B. Persson, Contact mechanics for randomly rough surfaces. Surf. Sci. Rep. **61**, 201–227 (2006).

[30] B. Persson , On the origin of Amonton’s friction law. J. Phys. Condens. Matter **20**, 395006 (2008).

[31] K. Obara, Nonvolcanic deep tremor associated with subduction in Southwest Japan. Science **296**, 1679–1681 (2002). 12040191 10.1126/science.1070378

[32] H. Dragert, K. L. Wang, T. S. James, A silent slip event on the deeper Cascadia subduction interface. Science **292**, 1525–1528 (2001). 11313500 10.1126/science.1060152

[33] A. Kato, S. Nakagawa, Detection of deep low-frequency earthquakes in the Nankai subduction zone over 11 years using a matched filter technique. Earth Planets Space **72**, 128 (2020).

[34] G. Farge, E. E. Brodsky, The big impact of small quakes on tectonic tremor synchronization. Sci. Adv. **11**, eadu7173 (2025). 40367185 10.1126/sciadv.adu7173 PMC12077523

[35] R. E. Mirollo, S. H. Strogatz, Synchronization of pulse-coupled biological oscillators. SIAM J. Appl. Math. **50**, 1645–1662 (1990).

[36] C. G. Sammis, S. W. Smith, Triggered tremor, phase-locking, and the global clustering of great earthquakes. Tectonophysics **589**, 167–171 (2013).

[37] W. Steinhardt, S. Dillavou, M. Agajanian, S. M. Rubinstein, E. E. Brodsky, Seismological stress drops for confined ruptures are invariant to normal stress. Geophys. Res. Lett. **50**, e2022GL101366 (2023).

[38] W. Steinhardt, E. E. Brodsky, Precursory locking precedes slip events on laboratory fault. Geophys. Res. Lett. **52**, e2024GL112882 (2025).

[39] R. E. Abercrombie, A. S. Baltay, Magnitude, depth, and methodological variations of spectral stress drop within the SCEC/USGS community stress drop validation study using the 2019 ridgecrest earthquake sequence. *Bull. Seismol. Soc. Am*. **115**, 2741–2768 (2025). 10.1785/0120250056.

[40] W. Brace, J. Byerlee, Stick-slip as a mechanism for earthquakes. Science **153**, 990–992 (1966). 17837252 10.1126/science.153.3739.990

[41] C. H. Scholz, Earthquakes and friction laws. Nature **391**, 37–42 (1998).

[42] J. R. Rice, Fault stress states, pore pressure distributions, and the weakness of the San Andreas fault. Int. Geophys. **51**, 475–503 (1992).

[43] G. Mollon, J. Aubry, A. Schubnel, Laboratory earthquakes simulations—Emergence, structure, and evolution of fault heterogeneity. J. Geophys. Res. Solid Earth **129**, e2023JB028626 (2024).

[44] C.-Y. Ke, G. C. McLaskey, D. S. Kammer, Rupture termination in laboratory-generated earthquakes. Geophys. Res. Lett. **45**, 12784–12792 (2018).

[45] F. P. Bowden, D. Tabor, The Friction and Lubrication of Solids (Oxford University Press, 1950).

[46] J. A. Greenwood, J. B. P. Williamson, Contact of nominally flat surfaces. Proc. R. Soc. Math. Phys. Eng. Sci. **295**, 300–319 (1966).

[47] E. Rabinowicz, Friction and Wear of Materials (Wiley, ed. 2, 1965).

[48] T. S. Majmudar, R. P. Behringer, Contact force measurements and stress-induced anisotropy in granular materials. Nature **435**, 1079–1082 (2005). 15973358 10.1038/nature03805

[49] D. Morad, A. Clark, E. E. Brodsky, “Local rheology of dense granular flow using a novel photoelastic couette-style deformation cell” in AGU Fall Meeting Abstracts (2024), p. EP23E-01.

[50] J. M. Fletcher, M. E. Oskin, O. J. Teran, The role of a keystone fault in triggering the complex El Mayor-Cucapah earthquake rupture. Nat. Geosci. **9**, 303–307 (2016).

[51] X. Guardiola, A. Díaz-Guilera, M. Llas, C. J. Pérez, Synchronization, diversity, and topology of networks of integrate and fire oscillators. Phys. Rev. E **62**, 5565–5570 (2000). 10.1103/physreve.62.5565 11089114

[52] P. Bak, C. Tang, Earthquakes as a self-organized critical phenomenon. J. Geophys. Res. Solid Earth **94**, 15635–15637 (1989).

[53] Z. Olami, H. J. S. Feder, K. Christensen, Self-organized criticality in a continuous, nonconservative cellular automaton modeling earthquakes. Phys. Rev. Lett. **68**, 1244–1247 (1992). 10046116 10.1103/PhysRevLett.68.1244

[54] P. Bak, C. Tang, K. Wiesenfeld, Self-organized criticality: An explanation of the 1/ f noise. Phys. Rev. Lett. **59**, 381–384 (1987). 10035754 10.1103/PhysRevLett.59.381

[55] H. Guo, T. Lay, E. E. Brodsky, Seismological indicators of geologically inferred fault maturity. J. Geophys. Res. Solid Earth **128**, e2023JB027096 (2023).

[56] A. Corral, F. Font-Clos, Criticality and self-organization in branching processes: application to natural hazards. arXiv [Preprint] (2012). https://arxiv.org/abs/1207.2589 (Accessed 4 December 2025).

[57] A. Helmstetter, Y. Kagan, D. Jackson, Importance of small earthquakes for stress transfers and earthquake triggering. J. Geophys. Res. **110**, B05S08 (2005).

[58] Y. Ogata, Statistical models for earthquake occurrences and residual analysis for point processes. J. Am. Stat. Assoc. **83**, 9–27 (1988).

[59] D. Sornette, M. J. Werner, Constraints on the size of the smallest triggering earthquake from the epidemic-type aftershock sequence model, Båth’s law, and observed aftershock sequences. J. Geophys. Res. **110**, 8304 (2005).

[60] B. M. Asayesh, S. Hainzl, G. Zöller, Improved aftershock forecasts using mainshock information in the framework of the ETAS model. J. Geophys. Res. Solid Earth **130**, e2024JB030287 (2025).

[61] J. McGuire, M. Boettcher, T. Jordan, Foreshock sequences and short-term earthquake predictability on East Pacific Rise transform faults. Nature **434**, 457–461 (2005). 15791246 10.1038/nature03377

[62] E. E. Brodsky , The state of stress on the fault before, during, and after a major earthquake. Annu. Rev. Earth Planet. Sci. **48**, 49–74 (2020).

[63] E. E. Brodsky, H. Kanamori, Elastohydrodynamic lubrication of faults. J. Geophys. Res. **106**, 16357–16374 (2001).

[64] M. G. Irmer, E. E. Brodsky, A. H. Clark, Granular temperature controls local rheology of vibrated granular flows. Phys. Rev. Lett. **134**, 048202 (2025). 39951600 10.1103/PhysRevLett.134.048202

[65] J. D. Kirkpatrick , The depth of pseudotachylyte formation from detailed thermochronology and constraints on coseismic stress drop variability. J. Geophys. Res. **117**, 06406 (2012).

[66] J. R. Rice, Heating and weakening of faults during earthquake slip. J. Geophys. Res. **111**, 2005JB004006 (2006).

[67] N. J. van der Elst, E. E. Brodsky, P.-Y. Le Bas, P. A. Johnson, Auto-acoustic compaction in steady shear flows: Experimental evidence for suppression of shear dilatancy by internal acoustic vibration. J. Geophys. Res. **117**, B09314 (2012).

[68] D. R. Faulkner , A review of recent developments concerning the structure, mechanics and fluid flow properties of fault zones. J. Struct. Geol. **32**, 1557–1575 (2010).

[69] T. Candela, E. E. Brodsky, C. Marone, D. Elsworth, Flow rate dictates permeability enhancement during fluid pressure oscillations in laboratory experiments. JGR Sold Earth **120**, 2037–2055 (2015).

[70] J. E. Elkhoury, E. E. Brodsky, D. C. Agnew, Seismic waves increase permeability. Nature **441**, 1135–1138 (2006). 16810253 10.1038/nature04798

[71] P. M. Fulton, E. E. Brodsky, In situ observations of earthquake-driven fluid pulses within the Japan Trench plate boundary fault zone. Geology **44**, 851–854 (2016).

[72] L. Xue , Continuous permeability measurements record healing inside the Wenchuan earthquake fault zone. Science **340**, 1555–1559 (2013). 23812711 10.1126/science.1237237

[73] M. Doan, E. E. Brodsky, Y. Kano, K. Ma, In situ measurement of the hydraulic diffusivity of the active Chelungpu Fault, Taiwan. Geophys. Res. Lett. **33**, L16317 (2006).

[74] H. Guo, E. E. Brodsky, T. H. W. Goebel, T. T. Cladouhos, Measuring fault zone and host rock hydraulic properties using tidal responses. Geophys. Res. Lett. **48**, e2021GL093986 (2021).

[75] L. Xue, E. E. Brodsky, J. Erskine, P. M. Fulton, R. Carter, A permeability and compliance contrast measured hydrogeologically on the San Andreas Fault. Geochem. Geophys. Geosyst. **17**, 858–871 (2016).

[76] J. A. Cherry, R. A. Freeze, Groundwater (Prentice-Hall, Englewood Cliffs, NJ, 1979).

[77] P. Talwani, L. Chen, K. Gahalaut, Seismogenic permeability, ks. J. Geophys. Res. Solid Earth **112**, 2006JB004665 (2007).

[78] P. M. Fulton, E. E. Brodsky, Determining hydraulic diffusivity from ambient noise in subsurface flow rate and temperature data. Geophys. J. Int. **234**, 2000–2006 (2023).

[79] K. Dascher-Cousineau, O. Shchur, E. E. Brodsky, S. Günnemann, Using deep learning for flexible and scalable earthquake forecasting. Geophys. Res. Lett. **50**, e2023GL103909 (2023).

[80] Y. Ogata, Statistics of earthquake activity: Models and methods for earthquake predictability studies. Annu. Rev. Earth Planet. Sci. **45**, 497–527 (2017).

